# Nitrous Oxyd Gas

**Published:** 1864-03

**Authors:** 


NITROUS OXYD GAS.—The following, which we clip from the *New York Times*, of March 12th, places rather a different phase upon the cases of death, reported to have been produced by Laughing Gas, and trumpeted over the country, with such apparent eagerness, without any investigation whatever.

Dr. Colton is a warm and earnest advocate of this anæsthetic, and while by some, his motives have been impugned, and his statements called in question, yet, we see no ground for the former, and can not indulge in the latter; but the correspondence which he here gives, from parties most competent to speak, in regard to these cases, will set the matter in a true light in the mind of every one. Let every one read the article.—ED.

LAUGHING GAS—AN ANÆSTHETIC.—Upon a thorough and impartial investigation, I have ascertained that the three deaths, reported as having been caused by the laughing gas, have no foundation in truth—that (except the first,) they originated in mere rumor, and that the paper publishing them, was imposed upon by inviduals interested in shaking public confidence in the gas as an anæsthetic agent. Now, what are the facts?

There have been only three deaths reported; Mr. Sears, in Canal street, of this City; Miss Bell, of Swanton, Vermont, and a lady at Allentown, Pennsylvania. Respecting the death of Mr. Sears, the *Medical and Surgical Reporter*

(highest authority in country) says: "From the pathological condition of the lungs of the patient. we have little doubt that the same result would have followed the extraction of a tooth, *if no anæsthetic had been taken.* When a person has so slight a hold on life as this man had, so insignificant a circumstance as the extraction of a tooth will often so derange the nervous and circulatory systems as to occasion the congestion which caused the death." This opinion is confirmed by every physician in this city with whom I have conversed on the subject. Certainly *this* death was not caused by the gas.

As to Miss Bell, of Swanton, Vt., in order to obtain correct information, I wrote a letter of inquiry to Dr. L. Gilman, of St. Alban's. He answers, under date of February 28: "Miss Bell was about 17, had been quite gay all Winter—had complained of headache some, but apparently in good health. She inhaled the *gas for sport*, with several others, on Friday afternoon, (Jan. 29,) came out of it as well as any one ever does, *attended a party that evening*, as well. to all appearance, as ever. The next morning she was taken or complained of her head and a difficulty with her throat." In another part of his letter, Dr. Gilman says: "She took the gas on *Friday*, attended a party the same evening, full of life and frolic as ever, was taken sick on Saturday and died the next *Wednesday*." These days and dates are corroborated by the St. Alban's *Messenger*, which calls her disease "inflammation of the meninges of the brain and spinal chord." No Coroner's Jury was summoned and the people of Swanton generally do not think the gas had anything to do with her death. Is it so strange a thing that one should die *five days* after being taken sick, that her death must be ascribed to the gas she had previously breathed "for sport?" In view of these facts, no rational person can ascribe this death to the laughing gas.

As to the lady in Allentown, Penn.—the third and last case—*she never breathed laughing gas at all*, but inhaled *chloroform*, and died from its effects. In order to get the facts respecting this Allentown case, I wrote a letter of inquiry to the Postmaster of that town, asking him to hand it to some good physician, with the request that he would give me the particulars. I received the following answer:

ALLENTOWN, Feb., 27, 1864.

DEAR SIR:—Your letter desiring information is received. An answer is most cheerfully given. *Chloroform* was the agent that produced the death in this case, which happened some time back, in the office of one of our most respectable dentists. It was administered, I believe, carefully, by two physicians of good standing in this place, and the quantity given not large, and the patient apparently healthy, which goes quite far to show that it is an agent that kills at times, and quite unforeseen and unexpected. Of late I have entirely refused to give it in the extraction of teeth.

Yours Respectfully,

C. L. MARTIN.

(In the country it is common for physicians to be called in to administer chloroform for dentists.)

Here the story is "made out of whole cloth," the lady never having breathed the nitrous oxide, but died from the effects of *chlcroform.* We see how easily these "reports" are dissipated by investigation.

We know that Sir Humphrey Davy often breathed this gas to the point of insensibility, and in his "Researches," published in 1799, the following remarkable passage occurs: "As nitrous oxyd in its extensive operation appears capable of destroying physical pain, it may probably be used with advantage in surgical operations"—a suggestion and prophesy which is now being fulfilled.

Prof. Benj. Silliman, in the last edition of his Chemistry, says of this gas: "*It may be breathed without injury*, but it produces a remarkable excitment in the system, amounting to intoxication, and, if carried far, to insensibility;" or to the anæsthetic state. All our works on chemistry speak of the gas as harmless.

Prof. R. Ogden Doremus, in his closing lecture on "*The World's indebtedness to Science*," said (TIMES report) "This gas was entirely harmless in its effects, and while a small dose would exhiliarate, a larger dose would induce anæsthesia or sound sleep, during which the most painful surgical operations could be performed without pain or the knowledge of the patient."

The March number of the *Dental Cosmos* contains an extract from the *Medical Times and Gazette*, giving the symptoms attending fifty-one cases of death from *chloroform*,

(not laughing gas,) in which the following passage occurs. "What is to be done in cases of threatened death? There is only one stimulus to the failing heart, the stimulus of areated blood; and the only means of producing this is by the excitation of respiration." A foot note at the bottom of the page, adds: "Arterialization and respiration can be most readily induced in asphyxia by *nitrous oyxd*, either in its gaseous form through the lungs, or condensed in water, and introduced into the alimentary canal by the mouth or bowels." Now that is capital! When a man is carried to a state of asphyxia, next to death's door, by *chloroform*, you can restore him to life by *nitrous oxyd!* And yet there is sound philosophy in the suggestion. The man dies, (if at all) not more from the direct effects of the chloroform, than from the lack of oxygen. Nitrous oxide contains *more* oxygen than the common air; being composed of the same elements (oxygen and nitrogen,) it contains one-third oxygen instead of one-fifth, as in the thin air. Oxgen is that which gives life to the blood and to the whole system. In giving chloroform you approach the point of death; the pulse gradually runs down, as also the breathing, until, if carried too far, they cease. A man can not live without oxygen, chloroform contains oxygen. The nitrous oxyd gives *life*, and by a few inhalations the blood, absorbing the gas readily, is started into circulation, and the man's life is saved. Will not every physician indorse this statement?

I have administered this gas as anæsthetic for the extraction of teeth, since May last, to thousands of patients, never failing to produce sleep and insensibility to pain, and have never had anything approaching ill effects to attend it. I am careful to make the gas pure, and administer it when fresh, and with discretion. A few days since, I administered it to a little boy 11 years of age, and, by alternating it with the air, kept him in a profound sleep for three minutes, during which time a surgeon extracted a piece of bone from the leg six inches in length. On waking, the little fellow asked, "When are you going to take out the bone?" I have no doubt I could have kept him asleep ten minutes.

From the fact that hundreds of dentists, who are not chemists, have made and admistered this gas for six or eight months past, *and not a single death has resulted*, is it not apparent that, in competent and experienced hands, it is by far the most safe anæsthetic known?—COLTON.

Growth of Bone.—M. de Lamballe appears, in the course of his extensive researches on the generation and reparation of tissues, to have corroborated the assertions of M. Flourens. (1.) That the bones increase in thickness by external and superimposed layers. (2.) That they increase in length by the addition of terminal layers arranged in juxtaposition. (3.) That proportionally as the new layers are deposited externally, the older ones on the inner surface are resorbed. (4.) That ossification consists in the regular and successive conversion of periosteum into cartilage, and of cartilage into bone.—*Popular Science Review.*

